# Integrated Care Using the ABC_stroke_ Pathway Improves Cardiovascular Outcomes and Survival in Patients with First-Ever Ischaemic Stroke

**DOI:** 10.5334/gh.1430

**Published:** 2025-05-27

**Authors:** Christopher T. W. Tsang, Sylvia E. Choi, Tommaso Bucci, Jia-Yi Huang, Qing-Wen Ren, Mei-Zhen Wu, Wen-Li Gu, Ran Guo, Jing-Nan Zhang, Anthony O. T. Ma, Steven H. M. Lam, Yap-Hang Chan, Kui-Kai Lau, Hung-Fat Tse, Azmil H. Abdul-Rahim, Gregory Y. H. Lip, Kai-Hang Yiu

**Affiliations:** 1 Cardiology Division, Department of Medicine, The University of Hong Kong, Hong Kong, China; 2 Liverpool Centre for Cardiovascular Science at University of Liverpool, Liverpool John Moores University and Liverpool Heart & Chest Hospital, Liverpool, UK; 3 Department of Cardiovascular and Metabolic Medicine, Institute of Life Course and Medical Sciences, University of Liverpool, Liverpool, UK; 4 Department of Clinical Internal, Anesthesiological and Cardiovascular Sciences, Sapienza University of Rome, Rome, Italy; 5 Cardiology Division, Department of Medicine, The University of Hong Kong Shen Zhen Hospital, Shen Zhen, China; 6 Neurology Division, Department of Medicine, The University of Hong Kong, Hong Kong, China; 7 State Key Laboratory of Brain and Cognitive Sciences, The University of Hong Kong, Hong Kong, China; 8 Stroke Division, Department of Medicine for Older People, Whiston Hospital, Mersey and West Lancashire Teaching Hospitals NHS Trust, Prescot, UK; 9 Danish Center for Health Services Research, Department of Clinical Medicine, Aalborg University, Denmark

**Keywords:** Stroke, post-stroke care, integrated care, ABC pathway, cardiovascular outcomes

## Abstract

**Background:**

A recent position paper of the European Society of Cardiology Council on Stroke proposed an integrated ABC_stroke_ pathway to optimise post-stroke management. We evaluated the impact of ABC_stroke_ pathway adherence on post-stroke cardiovascular outcomes.

**Methods:**

Patients with first-ever ischaemic stroke in Hong Kong between 2006 and 2022 were included in this retrospective cohort study. Multivariable Cox regression analysis was performed to evaluate the association between physicians’ adherence to the ABC_stroke_ pathway and the primary outcome, which was a composite of recurrent ischaemic stroke, transient ischaemic attack, haemorrhagic stroke, myocardial infarction, heart failure and all-cause mortality.

**Results:**

Of the 9,669 included patients with ischaemic stroke (mean age 69.6 ± 13.4 years; 57.5% male), 58.1% were optimally managed according to all three ABC_stroke_ pillars. After 1 year of follow-up, adherence to the ABC_stroke_ pathway was associated with a lower risk of the primary composite endpoint (hazard ratio (HR): 0.80; 95% confidence interval (CI): 0.72–0.88), as well as a lower risk of haemorrhagic stroke (subdistribution hazard ratio (SHR): 0.50; 95% CI: 0.38–0.67), heart failure (SHR: 0.771; 95% CI: 0.596–0.998), cardiovascular death (SHR: 0.64; 95% CI: 0.45–0.90), and all-cause mortality (HR: 0.72; 95% CI: 0.62–0.85). Risk reductions in the primary endpoint increased progressively with a higher number of ABC_stroke_ criteria obtained. No significant interaction was observed in the association according to age, sex, or stroke severity.

**Conclusions:**

In this cohort of Asian patients with first-ever ischaemic stroke, optimal management according to the ABC_stroke_ pathway was associated with a reduction in the risk of adverse outcomes.

## Introduction

Post-stroke cardiovascular complications, including acute myocardial injury, left ventricular dysfunction and cardiac arrhythmia, are common in patients with ischaemic stroke (IS), with an estimated incidence of around 20% (1,2). The term ‘Stroke-Heart Syndrome’ has been coined to describe the acute cardiac manifestations that result from IS (3,4). Recent epidemiological studies have shown that Stroke-Heart Syndrome is associated with unfavourable consequences such as recurrent strokes, secondary cardiac events, cognitive impairment, and death (1,5,6,7). Despite current preventive therapies, including antithrombotic treatment, blood pressure control, and lipid lowering, secondary prevention remains suboptimal among post-stroke patients (8,9), leaving them at increased risk of future adverse events. An integrated care approach is therefore warranted to reduce this residual risk.

In a recent position paper of the European Society of Cardiology (ESC) Council on Stroke, a holistic integrated care management for stroke patients was proposed (the ‘ABC_stroke_ pathway’) (10). The ABC_stroke_ pathway includes three pillars of management: (A) Appropriate antithrombotic therapy; (B) Better functional and psychological status; (C) Cardiovascular risk factors and comorbidity optimisation (including lifestyle changes). However, studies investigating the impact of physicians’ adherence to the ABC_stroke_ pathway on cardiovascular outcomes, especially in an Asian cohort, are scarce. By utilising a population-based cohort in Hong Kong, our study aimed to evaluate the impact of optimising integrated care for real-world patients with first-ever IS using the ABC_stroke_ pathway on cardiovascular outcomes.

## Methods

Data in this retrospective cohort study were retrieved from the Clinical Data Analysis and Reporting System (CDARS), an electronic health record database operated by the Hong Kong Hospital Authority. All diagnoses in CDARS are coded by the International Classification of Diseases, Ninth Revision, Clinical Modification (ICD-9-CM), which has previously been shown to have good coding accuracy (11).

This study was reported in accordance with the Strengthening the reporting of observational studies in epidemiology (STROBE) statement. As patient data was de-identified in CDARS, the need for individual consent was waived. The study has been approved by the Institutional Review Board of the University of Hong Kong/Hospital Authority Hong Kong West Cluster [Reference Number: UW 24–187].

### Study Population

**Figure 1:**
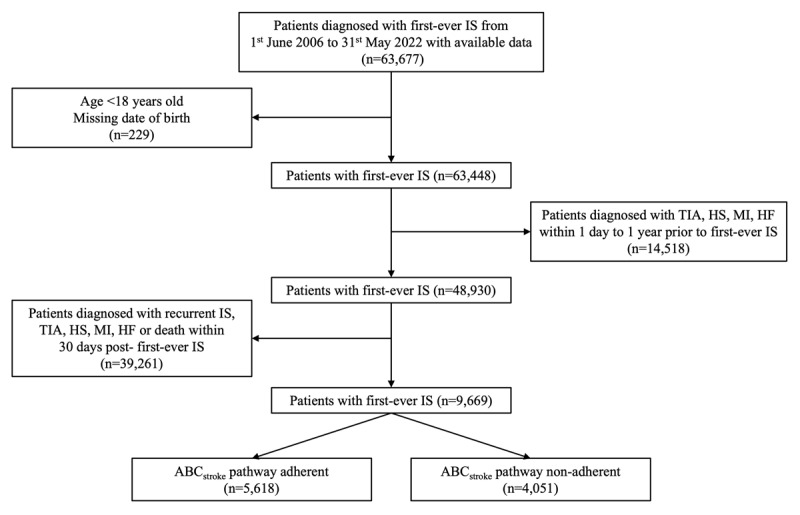
Flow chart of the study cohort. IS = ischaemic stroke; TIA = transient ischaemic attack; HS = haemorrhagic stroke; MI = myocardial infarction; HF = heart failure; mRS = modified Rankin scale; NIHSS = National Institutes of Health Stroke Scale.

Patients aged 18 years old or above with first-ever IS recorded in CDARS between 1^st^ June 2006 and 31^st^ May 2022 were included. The index date was defined as the date when a patient was diagnosed with IS for the first time. To allow the evaluation of incident transient ischaemic attack (TIA), haemorrhagic stroke (HS), myocardial infarction (MI) and heart failure (HF) as our study outcomes, patients with these diagnoses at baseline were excluded. Patients’ treatment prescriptions were evaluated within 30 days post-index date. Patients presenting with the study outcomes within 30 days post-index date were therefore excluded as their treatment prescriptions could not be fully ascertained. Patients with missing information on the modified Rankin scale (mRS) or National Institutes of Health Stroke Scale (NIHSS) were also excluded. The flow chart of the study cohort is summarised in Figure 1.

### Baseline information

Medical records of each patient were traced back to 1 year prior to the index date for evaluating baseline characteristics. The following data at baseline were collected: age, sex, smoking, alcohol use, NIHSS, comorbidities including atrial fibrillation (AF), hypertension (HTN), ischaemic heart disease (IHD), diabetes mellitus (DM), dyslipidaemia, chronic kidney disease (CKD), chronic liver disease (CLD), dementia, and medication prescriptions including angiotensin-converting enzyme inhibitors (ACEi), angiotensin receptor blockers (ARB), beta-blockers, calcium channel blockers (CCB), aspirin, P2Y12 inhibitors, warfarin, non-vitamin K antagonist oral anticoagulants (NOAC), insulin, metformin and statins. Baseline medication use was defined by filled prescription for at least 30 consecutive days prior to index date. Details of ICD-9-CM codes used for data collection are summarised in Table S1.

### Evaluation of the ABC_stroke_ pathway adherence

Physicians’ adherence to the ABC_stroke_ pathway was evaluated with reference to the position paper of the ESC Council on Stroke (10). The definition adopted in this study for each criterion is as follows:

‘A’ criterion: For appropriate antithrombotic therapy, patients with AF were adherent to the ‘A’ criterion if they had been prescribed oral anticoagulants either as warfarin or NOACs after stroke. For patients without AF, they were adherent if appropriate antiplatelet therapy such as aspirin or P2Y12 inhibitor was prescribed.

‘B’ criterion: To be adherent with the ‘B’ criterion, all stroke patients with any deficit at discharge, defined based on the mRS >2, should have been prescribed with stroke rehabilitation. Patients with a mRS ≤2 would also qualify for the ‘B’ criterion.

‘C’ criterion: For cardiovascular risk factors and comorbidities optimisation, we considered the use of statins and management of HTN, IHD and DM. Optimal medical treatment for the listed comorbidities was defined as follows: 1) for HTN, treatment with monotherapy or combination therapy of ACEi/ARB, CCB, or diuretics; 2) for IHD, treatment with ACEi/ARB, and beta-blocker; 3) for DM, treatment with insulin or metformin. Patients were considered adherent to the ‘C’ criterion when all comorbidities were properly treated, and a statin was prescribed.

Patients that were managed according to all three of the ABC_stroke_ criteria were considered to be in the ABC_stroke_ adherent group while patients with at least one of the ABC_stroke_ criteria not attained were in the ABC_stroke_ non-adherent group.

### Study outcomes

Patients were followed-up for up to 1 year. The primary endpoint of this study was a composite outcome of recurrent IS, incident TIA, incident HS, incident MI, incident HF and all-cause mortality. Secondary outcomes were the individual components of the composite outcome, and cardiovascular death.

### Statistical analyses

Continuous variables with normal distribution were reported as mean ± standard deviation while variables with non-normal distribution were reported as median [interquartile range]. Categorical variables were reported as absolute numbers and percentages. Differences between groups were compared using the independent sample t-test for continuous variables and the chi-squared test for categorical variables.

Cox proportional hazards regression models were used to calculate the hazard ratios (HR) with 95% confidence intervals (CI) to evaluate the association between adherence to the ABC_stroke_ pathway and the study outcomes. The multivariable Cox model was adjusted for age at index date, sex, smoking, alcohol use, NIHSS at admission, baseline comorbidities (AF, HTN, IHD, DM, dyslipidaemia, CKD, CLD, dementia), and baseline medication use (ACEi, ARB, beta-blocker, CCB, antiplatelets, anticoagulants, antidiabetics, statins). To account for competing risk, the Fine-Gray model was used to calculate the subdistribution hazard ratio (SHR) and the corresponding 95% CI for the risk of the secondary outcomes, with all-cause death defined as the competing event. To evaluate if a greater number of the ABC_stroke_ criteria attained was associated with a progressive risk reduction, we investigated the risk of the primary outcome as stratified by the number of ABC_stroke_ criteria attained, with patients attaining zero or one criterion as the reference group. Additionally, we investigated the effect of each ABC_stroke_ criterion in the outcomes of interest by including each criterion as a different variable in the multivariable Cox and Fine-Gray models. Differences in Kaplan-Meier curves between ABC_stroke_ adherent versus ABC_stroke_ non-adherent groups, and patients with different numbers of ABC_stroke_ criteria attained were evaluated with the log-rank tests.

Subgroup analyses were performed for the primary outcome using the multivariable Cox model for clinically relevant variables including age, sex, NIHSS, year of stroke diagnosis, and baseline comorbidities (HTN and DM). Two sensitivity analyses were conducted to ascertain the robustness of our findings. First, we performed conventional Cox regression analyses without competing risk. Second, we used the inverse probability of treatment weighting (IPTW) to further adjust for confounders. All baseline covariates of each individual were logistically regressed to calculate the probability of receiving the interventions for ABC_stroke_ pathway adherence, that is their propensity score. IPTW creates a pseudo-population by assigning individuals with weights that correspond to the inverse of their propensity scores, such that confounders are equally distributed between the ABC_stroke_ adherent and ABC_stroke_ non-adherent groups (12). After applying IPTW, baseline characteristics were considered well-balanced between the ABC_stroke_ adherent and ABC_stroke_ non-adherent groups if the standardised mean differences were ≤0.1. All statistical analyses were performed using R, version 4.3.1, The R Foundation, 2023. A two-way *P*-value <0.05 was considered to be statistically significant.

## Results

### Study cohort

**Figure 2:**
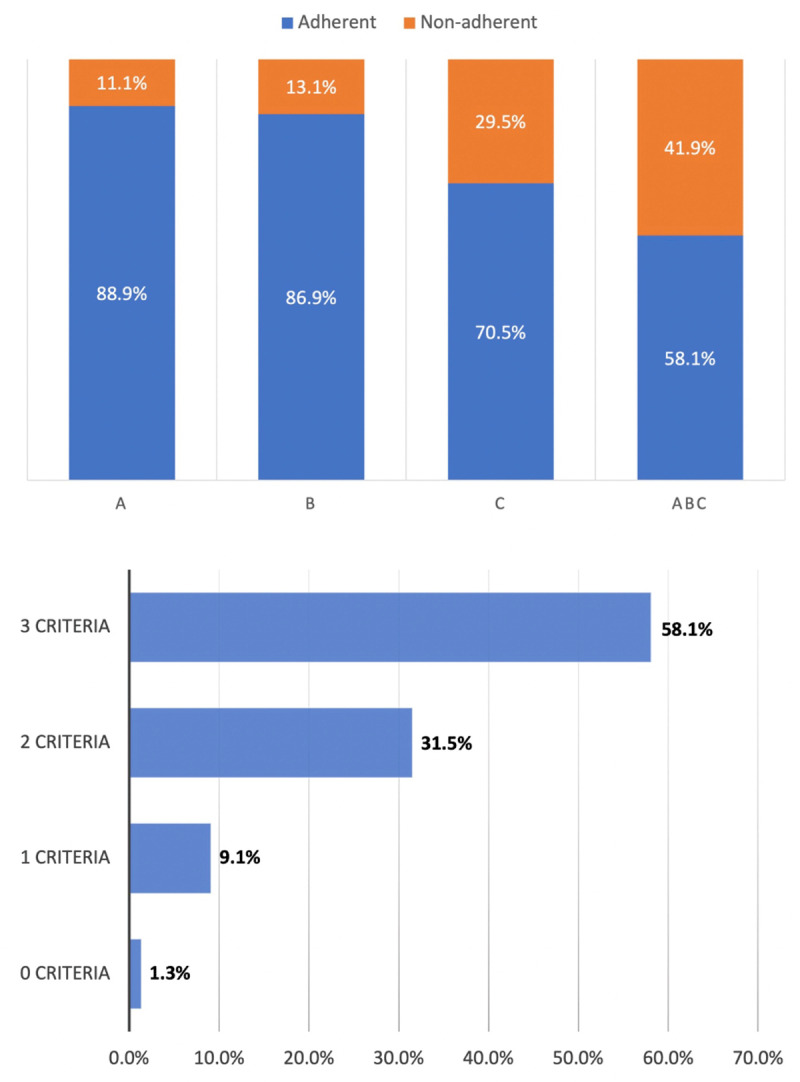
Distribution of ABC_stroke_ criteria adherence and the number of ABC_stroke_ criteria attained.

A total of 9,669 eligible patients diagnosed with first-ever IS were included in this analysis (Figure 1). The mean age was 69.6 ± 13.4 years and 5,560 (57.5%) patients were male. The median NIHSS was 3 [1–6], representing mildly severe strokes (Table 1). Among those patients, 5,618 (58.1%) were fully ABC_stroke_ adherent and the remaining 4,051 (41.9%) were ABC_stroke_ non-adherent. In this final cohort, 8,595 (88.9%) patients were managed according to the ‘A’ criterion, 8,404 (86.9%) to the ‘B’ criterion, and 6,821 (70.5%) to the ‘C’ criterion. The number of patients with zero, one, or two criteria attained is reported in Figure 2.

**Table 1:** Baseline characteristics of ABC_stroke_ adherent and non-adherent patients.


	ALL PATIENTS (n = 9,669)	ABC ADHERENT (n = 5,618)	ABC NON-ADHERENT (n = 4,051)	*p* VALUE

Age (years)	69.6 ± 13.4	67.9 ± 12.7	72.0 ± 14.0	<0.001

Male	5,560 (57.5)	3,336 (59.4)	2,224 (54.9)	<0.001

Smoking	3,276 (33.9)	2,026 (36.1)	1,250 (30.9)	<0.001

Alcohol	1,908 (19.7)	1,186 (21.1)	722 (17.8)	<0.001

NIHSS	3 [1–6]	3 [1–5]	4 [1–9]	<0.001

Baseline comorbidities				

Atrial fibrillation	222 (2.3)	85 (1.5)	137 (3.4)	<0.001

Hypertension	812 (8.4)	442 (7.9)	370 (9.1)	0.029

Ischaemic heart disease	154 (1.6)	73 (1.3)	81 (2.0)	0.009

Diabetes mellitus	498 (5.2)	254 (4.5)	244 (6.0)	0.001

Dyslipidaemia	249 (2.6)	155 (2.8)	94 (2.3)	0.201

Chronic kidney disease	98 (1.0)	42 (0.7)	56 (1.4)	0.003

Chronic liver disease	49 (0.5)	20 (0.4)	29 (0.7)	0.021

Dementia	196 (2.0)	52 (0.9)	144 (3.6)	<0.001

Baseline medication use				

ACEi	1,543 (16.0)	859 (15.3)	684 (16.9)	0.037

ARB	536 (5.5)	329 (5.9)	207 (5.1)	0.124

Beta-blocker	2,018 (20.9)	1,052 (18.7)	966 (23.8)	<0.001

CCB	3,016 (31.2)	1,684 (30.0)	1,332 (32.9)	0.003

Aspirin	1,567 (16.2)	773 (13.8)	794 (19.6)	<0.001

P2Y12 inhibitor	90 (0.9)	43 (0.8)	47 (1.2)	0.059

Warfarin	108 (1.1)	34 (0.6)	74 (1.8)	<0.001

NOAC	70 (0.7)	25 (0.4)	45 (1.1)	<0.001

Insulin	325 (3.4)	191 (3.4)	134 (3.3)	0.849

Metformin	1,370 (14.2)	833 (14.8)	537 (13.3)	0.031

Statin	1,849 (19.1)	1,204 (21.4)	645 (15.9)	<0.001


Values are shown as mean ± standard deviation, median [interquartile range], or n (%). NIHSS = National Institutes of Health Stroke Scale; ACEi = angiotensin-converting enzyme inhibitor; ARB = angiotensin receptor blocker; CCB = calcium channel blocker; NOAC = non-vitamin K antagonist oral anticoagulants.

As reported in Table 1, ABC_stroke_ non-adherent patients were older, less likely to be male, and had a higher NIHSS on admission compared to patients who were ABC_stroke_ adherent. In addition, ABC_stroke_ non-adherent patients more often had a medical history of AF, HTN, IHD, DM, CKD, CLD and dementia than ABC_stroke_ adherent patients. A summary of the clinical characteristics is shown in Table 1 and Table S2.

### Outcomes and survival analysis

At 1 year follow-up, 2,080 adverse cardiovascular events and deaths were recorded. Compared with patients who were ABC_stroke_ non-adherent, patients who were optimally managed according to all three ABC_stroke_ criteria had a lower incidence of the primary composite outcome (13.9% vs 22.2%; P < 0.001) (Table 2).

**Table 2:** Effect of ABC_stroke_ pathway adherence on the risk for adverse cardiovascular events and death.


	EVENT NUMBER (%)	UNADJUSTED HR/SHR (95% CI)	*p* VALUE	ADJUSTED HR/SHR (95% CI)	*p* VALUE

**Composite outcome**					

ABC adherent	783 (13.9)	0.59 (0.54–0.65)	<0.001	0.80 (0.72–0.88)	<0.001

ABC non-adherent	899 (22.2)	Ref.		Ref.	

Recurrent IS*					

ABC adherent	331 (5.9)	0.95 (0.81–1.12)	0.570	1.00 (0.84–1.19)	0.990

ABC non-adherent	250 (6.2)	Ref.		Ref.	

TIA*					

ABC adherent	71 (1.3)	1.35 (0.91–2.00)	0.140	1.29 (0.86–1.95)	0.220

ABC non-adherent	38 (0.9)	Ref.		Ref.	

HS*					

ABC adherent	83 (1.5)	0.38 (0.29–0.49)	<0.001	0.50 (0.38–0.67)	<0.001

ABC non-adherent	157 (3.9)	Ref.		Ref.	

MI*					

ABC adherent	70 (1.2)	0.90 (0.63–1.28)	0.560	1.16 (0.79–1.70)	0.440

ABC non-adherent	56 (1.4)	Ref.		Ref.	

HF*					

ABC adherent	124 (2.2)	0.54 (0.43–0.68)	<0.001	0.77 (0.60–1.00)	0.048

ABC non-adherent	165 (4.1)	Ref.		Ref.	

Cardiovascular death*					

ABC adherent	60 (1.1)	0.44 (0.32–0.61)	<0.001	0.64 (0.45–0.90)	0.011

ABC non-adherent	97 (2.4)	Ref.		Ref.	

**All-cause mortality**					

ABC adherent	280 (5.0)	0.43 (0.37–0.50)	<0.001	0.72 (0.62–0.85)	<0.001

ABC non-adherent	455 (11.2)	Ref.		Ref.	


IS = ischaemic stroke; TIA = transient ischaemic attack; HS = haemorrhagic stroke; MI = myocardial infarction; HF = heart failure; HR = hazard ratio; SHR = subdistribution hazard ratio; CI = confidence interval. * = Fine-Gray model was used to adjust for competing risk, with death being the competing event.

**Figure 3:**
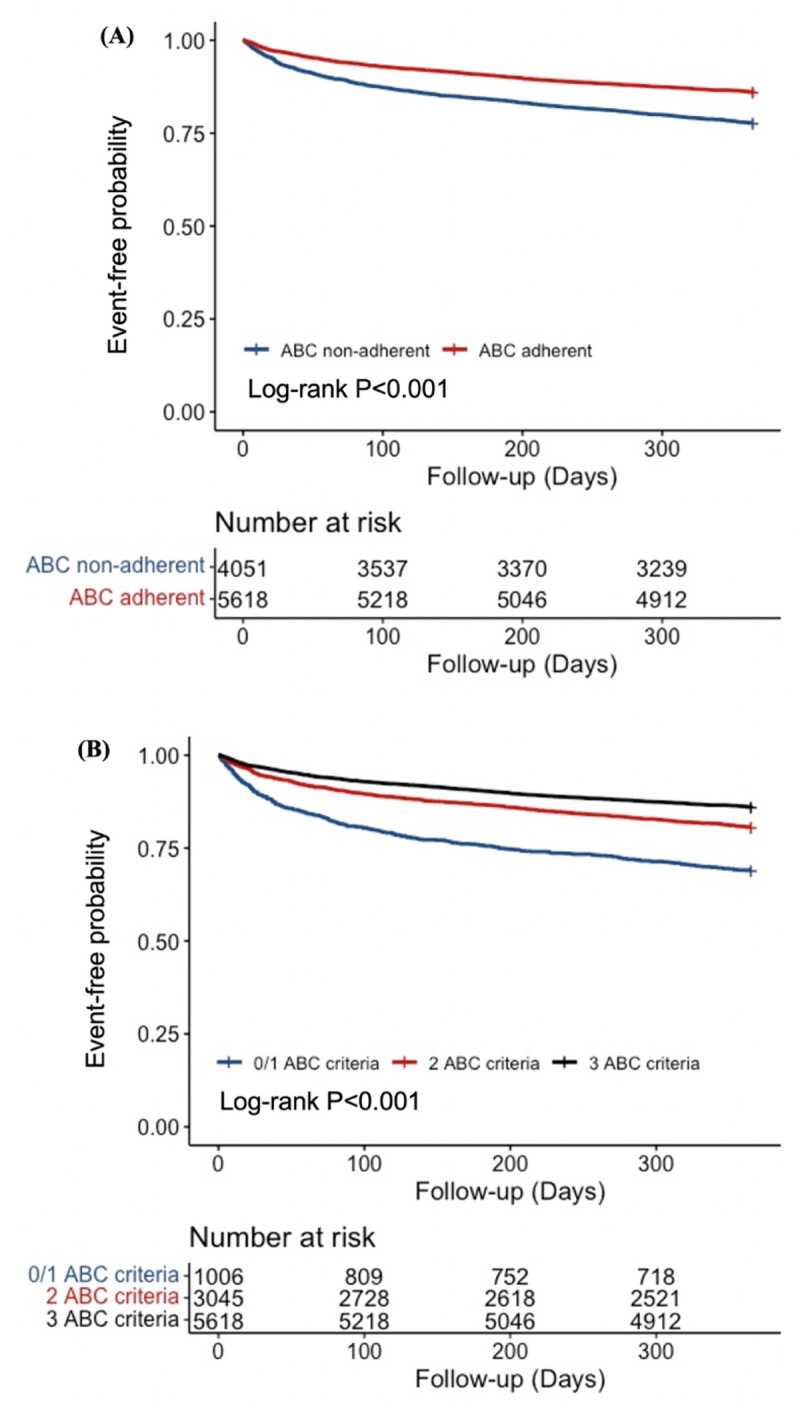
Kaplan-Meier curve showing **(A)** the risk of composite outcome between ABC_stroke_ adherent and ABC_stroke_ non-adherent patients; and **(B)** the risk of composite outcome in patients with 0 or 1 ABC_stroke_ criteria compared with patients with 2 or 3 ABC_stroke_ criteria attained.

On multivariable Cox regression analysis, full adherence to the ABC_stroke_ pathway was associated with a 20% risk reduction for the occurrence of the primary cardiovascular composite outcome (HR: 0.80; 95% CI: 0.72–0.88), as well as a significantly lower risk of all-cause mortality (HR: 0.72; 95% CI: 0.62–0.85), as visualised in the Kaplan-Meier curve in Figure 3A. Accounting for the competing risk of all-cause death, ABC_stroke_ pathway-adherent care was associated with a reduced risk of HS (SHR: 0.50; 95% CI: 0.38–0.67), HF (SHR: 0.771; 95% CI: 0.596–0.998), and cardiovascular death (SHR: 0.64; 95% CI: 0.45–0.90). No significant associations were found between ABC_stroke_ adherence and other secondary outcomes (Table 2).

Moreover, the multivariable Cox regression and Kaplan-Meier analyses showed a progressive risk reduction for the primary composite endpoint in patients who attained two ABC_stroke_ criteria (HR: 0.74; 95% CI: 0.64–0.85) and three ABC_stroke_ criteria (HR: 0.64; 95% CI: 0.55–0.73), compared to only zero or one ABC_stroke_ criteria (Figure 3B, Table S3).

Regarding the effect of each individual ABC_stroke_ criterion, adherence to A criterion was associated with significant reduction of the risk of composite outcome (HR: 0.60; 95% CI: 0.53–0.68), HS (HR: 0.20; 95% CI: 0.15–0.26), HF (HR: 0.65; 95% CI: 0.48–0.88), cardiovascular death (HR: 0.55; 95% CI: 0.37–0.81), and all-cause mortality (HR: 0.69; 95% CI: 0.58–0.83). Adherence to B criterion was associated with significant reduction of the risk of all-cause mortality (HR: 0.80; 95% CI: 0.67–0.95). Finally, adherence to C criterion was associated with significant reduction of the risk of composite outcome (HR: 0.85; 95% CI: 0.77–0.95), HS (HR: 0.62; 95% CI: 0.48–0.81), cardiovascular death (HR: 0.60; 95% CI: 0.43–0.85), and all-cause mortality (HR: 0.84; 95% CI: 0.72–0.98) (Table 3).

**Table 3:** Effect of adherence to each component of the ABC_stroke_ pathway on the risk for adverse cardiovascular events and death.


	CRITERION	ADJUSTED HR/SHR (95% CI)

**Composite outcome**	A	0.60 (0.53–0.68)

	B	0.97 (0.85–1.10)

	C	0.85 (0.77–0.95)

Recurrent IS*	A	0.99 (0.77–1.28)

	B	1.13 (0.87–1.47)

	C	1.00 (0.83–1.20)

TIA*	A	1.62 (0.74–3.54)

	B	1.18 (0.61–2.27)

	C	1.10 (0.70–1.71)

HS*	A	0.20 (0.15–0.26)

	B	1.38 (0.93–2.03)

	C	0.62 (0.48–0.81)

MI*	A	0.97 (0.56–1.67)

	B	0.96 (0.59–1.57)

	C	1.09 (0.73–1.64)

HF*	A	0.65 (0.48–0.88)

	B	0.91 (0.67–1.26)

	C	0.82 (0.63–1.06)

Cardiovascular death*	A	0.55 (0.37–0.81)

	B	0.95 (0.63–1.42)

	C	0.60 (0.43–0.85)

**All-cause mortality**	A	0.69 (0.58–0.83)

	B	0.80 (0.67–0.95)

	C	0.84 (0.72–0.98)


IS = ischaemic stroke; TIA = transient ischaemic attack; HS = haemorrhagic stroke; MI = myocardial infarction; HF = heart failure; HR = hazard ratio; SHR = subdistribution hazard ratio; CI = confidence interval. * = Fine-Gray model was used to adjust for competing risk, with death being the competing event.

### Subgroup analysis

**Figure 4:**
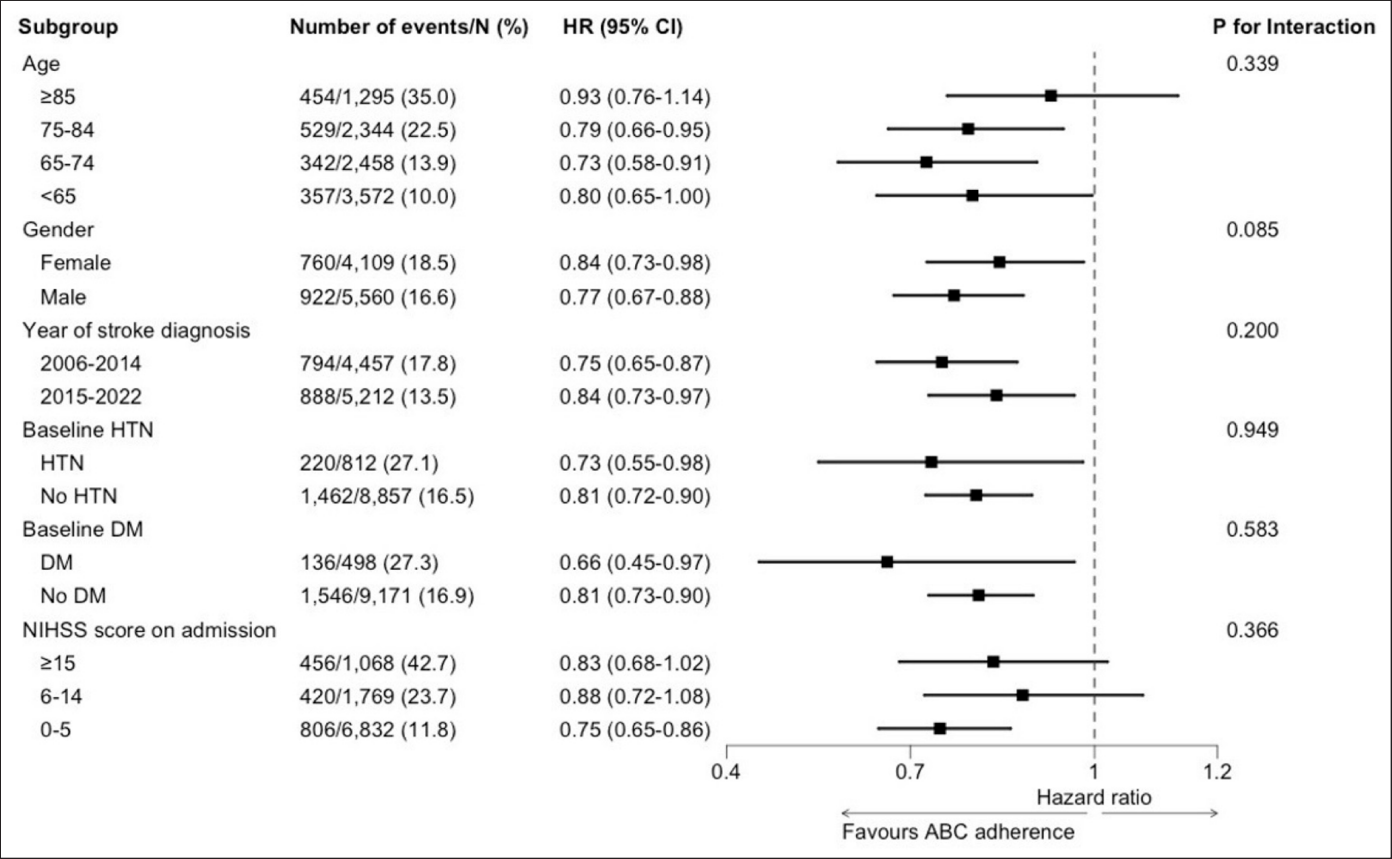
Subgroup analysis for the risk of composite outcome in different subgroups. HTN = hypertension; DM = diabetes mellitus; NIHSS = National Institutes of Health Stroke Scale; HR = hazard ratio; CI = confidence interval.

A similar effect size was observed in patients from different age groups, female or male patients, patients diagnosed in years 2006–14 and 2015–22, patients with or without HTN or DM, and patients with different NIHSS on admission (Figure 4). ABC_stroke_ pathway adherent care was associated with a reduced risk of the composite outcome, regardless of age, gender, year of stroke diagnosis, baseline HTN or DM, and stroke severity based on NIHSS on admission.

### Sensitivity analysis

We performed conventional Cox regression analysis of the secondary outcomes without adjusting for competing risk. In keeping with the main analysis, ABC_stroke_ pathway adherent care was associated with lower risk of HS, HF, and cardiovascular death compared with patients non-adherent to the ABC_stroke_ pathway (Table S4). We further adopted a Cox regression analysis of the primary outcome with IPTW. After matching with IPTW, baseline characteristics were well balanced between the ABC_stroke_ adherent group and the ABC_stroke_ non-adherent group (Table S5). Consistent with the main analysis, multivariable analysis after IPTW showed that the ABC_stroke_ adherent group had a lower risk of the primary composite endpoint (HR: 0.73; 95% CI: 0.66–0.80), compared with the ABC_stroke_ non-adherent group (Table S6).

## Discussion

In this population-based retrospective cohort study of patients with first-ever IS, our main findings are as follows: (i) 58.1% of the cohort were managed with full adherence to the three pillars of the ABC_stroke_ pathway; (ii) optimal management according to the ABC_stroke_ pathway was associated with a 20% risk reduction for the occurrence of the composite outcome, as well as a lower risk of HS, HF, cardiovascular death, and all-cause mortality at 1 year follow-up; and (iii) full adherence to the ABC_stroke_ pathway was associated with a lower risk of the composite outcome regardless of age, sex or stroke severity.

Beyond its recent implementation in stroke patients, the ABC pathway integrated care concept has been utilised in the management of various chronic long-term conditions. For example, in AF, the Atrial fibrillation Better Care pathway has been widely validated (13,14,15,16,17,18). Having been shown to be associated with improved cardiovascular outcomes, it is now adopted in various international guidelines on the management of AF (19,20,21).

Notably, we found in this study that ABC_stroke_ non-adherent patients were more likely to be female, older, and have medical comorbidities such as AF and DM. Interestingly, studies that investigated the Atrial fibrillation Better Care pathway in AF patients also reported that patients not in the integrated care group were more likely to be female (14,17) or older (14,18). As indicated by a recent study, higher stroke incidence among females with AF may stem from advanced age and disparities in cardiovascular healthcare (22). Our study provides further support for this notion, highlighting age and sex-based inequities in post-stroke management. While treatment choices should be individualised based on patients’ conditions, our subgroup analysis highlighted that optimal treatment according to the ABC_stroke_ pathway was associated with a lower risk of the composite cardiovascular outcome regardless of age, gender, baseline HTN or DM, and stroke severity, thus advocating for the adoption of this integrated care approach in a broader context. Importantly, cardiovascular risk factors and comorbidities optimisation for the ‘C’ criterion was achieved in only 70.5% of this cohort. This suboptimal level of vascular risk factor control in post-stroke patients was also reported in the EUROASPIRE III survey (8) and the National Health and Nutrition Examination Survey (9). These real-world data highlight important treatment gaps for targeted interventions to further increase the physicians’ adherence rate of managing these modifiable risk factors. It is worth noting that the components in this ABC_stroke_ pathway are not absolute recommendations. The essence of this integrated care pathway is to recognise and optimise these comorbidities with the latest guideline-directed medical therapy in a patient-centred approach, taking each patient’s preference, values, and co-morbid conditions into account.

Stroke-Heart Syndrome poses a considerable health challenge in the management of acute IS, yet there remains a lack of data to guide its prevention and treatment (2,3,23,24,25). In this representative cohort, we found that 17% of the post-stroke patients had at least one of the adverse cardiovascular composite outcomes at 1 year follow-up and over one-fifth of deaths were due to cardiovascular causes. These are comparable to findings from previous studies (1,2,6,26,27), which reported post-stroke adverse cardiac outcomes in 5–28% of patients, highlighting the importance of optimising management strategies for individuals with recent stroke and concomitant heart disease. Our findings complement and support recent observations in a study conducted in the Athens Stroke Registry (28), a single-centre study that included 2,513 patients admitted with acute first-ever IS between 1992 and 2012. Over a median follow-up period of 30 months, the authors concluded that adherence to the ABC_stroke_ pathway was associated with lower risks of stroke recurrence, major cardiovascular events and mortality (28). In our study, ABC_stroke_ pathway adherent care was associated with a more modest risk reduction in the composite of cardiovascular outcomes (20% in this study vs 41% in the Athens study) and the association with recurrent IS did not reach statistical significance. Besides potential differences in baseline characteristics, this discrepancy in the results may be due to the very low proportions of ABC_stroke_ adherent patients in the Athens study. Our study, which involved a more contemporary cohort, revealed a significantly higher proportion of patients (58.1%) who attained full adherence to the ABC_stroke_ pathway, in contrast to the Athens study, in which only 6.2% of patients achieved the same level of adherence. This discrepancy may be attributable to the low prescription rate of statins in post-stroke patients observed in the Athens study, where many patients were diagnosed with stroke much earlier than the 2006 Stroke Prevention and Aggressive Reduction in Cholesterol Levels (SPARCL) randomised trial that established the benefits of statin treatment in the secondary prevention of non-cardioembolic stroke (29). Indeed, only a small proportion of patients in the Athens study were treated with statins on discharge (20.1% in the Athens study vs 78.6% in this study), thus the remaining majority of patients would have been regarded as ABC_stroke_ non-adherent (in particular, with respect to criterion ‘C’). Furthermore, we included a larger patient sample and employed an extensive multivariable model, integrating key demographics, comorbidities, and medications, and utilised the Fine-Gray model to address competing risks in individual secondary outcomes. Our study demonstrated that optimal treatment according to the ABC_stroke_ pathway was independently associated with reduced risk of HS, HF, cardiovascular death, and all-cause mortality, with a progressively lower risk observed as higher numbers of ABC_stroke_ criteria were achieved. Of note, the reduction in risk of cardiovascular composite outcome was likely attributable to the effect of appropriate antithrombotic therapy and comorbidity optimisation (i.e. adherence to criteria ‘A’ and ‘C’) while appropriate stroke rehabilitation (i.e. adherence to criterion ‘B’) was associated with reduced risk of all-cause mortality. Therefore, while partial adherence to the ABC_stroke_ pathway may confer some clinical benefit, our findings suggest that reinforcing full adherence is crucial in achieving optimal clinical outcomes.

To improve the awareness and management of Stroke-Heart Syndrome as a distinct entity and its associated outcomes, the need for multidisciplinary clinical and research collaborations is crucial. Overall, a holistic and comprehensive, integrated approach to post-stroke care, as promoted in the ABC_stroke_ pathway, can deliver multifaceted, streamlined and coordinated action to target all areas of stroke management.

### Limitations

We acknowledge some potential limitations of our data and its interpretation. First, due to the retrospective and observational nature of this study, causality cannot be established and there may be residual confounding despite our use of IPTW in the sensitivity analysis to balance the clinically relevant variables between the two groups. For instance, data on educational level, socioeconomic status, and lifestyle factors were not available in CDARS, which may have led to bias in the results. Second, diagnoses rely on the accuracy of recording of ICD codes and may be subject to over- or under-reporting. Third, we included only patients with available data regarding mRS and NIHSS, and thus excluded quite a significant proportion of the study population as these are not routinely entered data. We found that patients with complete information of these data were younger, more likely to be male, smokers, drinkers, and had fewer baseline comorbidities and medication use (Table S7). Our findings may therefore have potential selection biases. Fourth, findings from this study are mostly restricted to Asian patients and hence our results may not be generalisable to other ethnic groups. Fifth, physicians’ adherence to initiating treatments in accordance with the ABC_stroke_ pathway was assessed within 30 days post-stroke, thus compliance and treatment changes that may have occurred during follow-up would not be captured. Lastly, the effect of the extent of optimisation during follow-up was not assessed and future clinical trials or prospective studies may incorporate surrogate markers of optimisation (e.g. lipids levels, blood pressure, haemoglobin A1c levels) for further evaluation.

## Conclusion

In this cohort of Asian patients with first-ever IS, optimal management according to the ABC_stroke_ pathway was evident in 58.1% of the cohort, and it was associated with significant risk reductions for the composite of cardiovascular outcomes, as well as for HS, HF, cardiovascular death and all-cause mortality. The beneficial effect observed was greatest among patients managed as fully adherent to ABC_stroke_ pathway, regardless of age, sex, or stroke severity. The findings in our study lend support to efforts to promote a holistic and integrated care approach to post-stroke management.

## Data Accessibility Statement

The data contains confidential information and hence cannot be shared with the public due to third-party use restrictions. Local academic institutions, government departments, or non-governmental organizations may apply for the access to data through the Hospital Authority’s data-sharing portal (https://www3.ha.org.hk/data).

## Additional File

The additional file for this article can be found as follows:

10.5334/gh.1430.s1 Supplementary Materials. Tables S1 to S8.
