# Supplementary material for: Integrated Care Using the ABCstroke Pathway Improves Cardiovascular Outcomes and Survival in Patients with First-Ever Ischaemic Stroke
Source: Glob Heart. 2025 May 27;20(1):46. doi: 10.5334/gh.1430 (PMC12124245; doi:10.5334/gh.1430)
Supplement: Supplementary Materials. — Tables S1 to S8. [file gh-20-1-1430-s1.pdf]

SUPPLEMENTARY MATERIALS

Table S1. ICD-9-CM codes used for the definition of covariates

Covariates	ICD-9-CM codes
Ischaemic stroke	433, 434, 436
Transient ischaemic attack	435
Haemorrhagic stroke	430, 431, 432<br>(Cases will be excluded if it is due to traumatic brain injury: 800-804, 850-854)
Myocardial infarction	410
Heart failure	398.91, 402.01, 402.11, 402.91, 404.01, 404.03, 404.11, 404.13, 404.91, 404.93, 428
Atrial fibrillation	427.31
Hypertension	401-405, 437.2
Ischaemic heart disease	410-414
Diabetes mellitus	250
Dyslipidaemia	272, 272.1, 272.2, 272.3, 272.4
Chronic kidney disease	585
Chronic liver disease	571-573
Dementia	290, 291.2, 292.82, 294.1, 331.0, 331.1, 331.3
Smoking	491, 492, 496
Alcohol use	291, 303, 305.0, 571.0, 571.1, 571.2, 571.3, E980.8, E980.9

ICD-9-CM = International Classification of Diseases, Ninth Revision, Clinical Modification.

Table S2. Characteristics and treatments of ABC_{stroke} adherent and non-adherent patients within 30 days post-stroke

	**All patients (n=9,669)**	**ABC adherent (n=5,618)**	**ABC non-adherent (n=4,051)**
Comorbidities			
Atrial fibrillation	831 (8.6)	328 (5.8)	503 (12.4)
Hypertension	3,733 (38.6)	2,019 (35.9)	1,714 (42.3)
Ischaemic heart disease	313 (3.2)	79 (1.4)	234 (5.8)
Diabetes mellitus	1,715 (17.7)	837 (14.9)	878 (21.7)
Symptomatic status			
mRS = 0	1,530 (15.8)	1,031 (18.4)	499 (12.3)
mRS = 1	3,166 (32.7)	2,166 (38.6)	1,000 (24.7)
mRS = 2	1,465 (15.2)	1,012 (18.0)	453 (11.2)
mRS = 3	890 (9.2)	252 (4.5)	638 (15.7)
mRS = 4	1,555 (16.1)	755 (13.4)	800 (19.7)
mRS = 5	1,063 (11.0)	402 (7.2)	661 (16.3)
Treatment			
OACs	522/831 (62.8)	328/328 (100)	194/503 (38.6)
Antiplatelets	8,073/8,838 (91.3)	5,290/5,290 (100)	2,783/3,548 (78.4)
Stroke rehabilitation for mRS>2 (n=3,508)	2,243/3,508 (63.9)	1,409/1,409 (100)	834/2,099 (39.7)
Statin	7,596/9,669 (78.6)	5,618/5,618 (100)	1,978/4,051 (48.8)
Antihypertensives	3,175/3,733 (85.1)	2,019/2,019 (100)	1,156/1,714 (67.4)
ACEi/ARB + Beta-blocker	108/313 (34.5)	79/79 (100)	29/234 (12.4)
Antidiabetics	1,325/1,715 (77.3)	837/837 (100)	488/878 (55.6)

Values are shown as n (%).

mRS = modified Rankin scale; OACs = oral anticoagulants; ACEi = angiotensin-converting enzyme inhibitor; ARB = angiotensin receptor blocker.

Table S3. Multivariable Cox regression analysis for the risk of composite outcome as stratified by the number of ABC_{stroke} criteria attained

	**HR**	**95% CI**	***P* value**
0-1 ABC criteria	Ref.	-	-
2 ABC criteria	0.74	0.64-0.85	<0.001
3 ABC criteria	0.64	0.55-0.73	<0.001

HR = hazard ratio; CI = confidence interval.

Table S4. Sensitivity analysis for the effect of ABC_{stroke} criteria adherence on the risk for adverse cardiovascular events and death on Cox-Regression analysis without competing risk

	**Event number (%)**	**Unadjusted HR (95% CI)**	***P* value**	**Adjusted HR (95% CI)**	***P* value**
**Composite outcome**					
ABC adherent	783 (13.9)	0.59 (0.54-0.65)	<0.001	0.80 (0.72-0.88)	<0.001
ABC non-adherent	899 (22.2)	Ref.		Ref.	
**Recurrent IS**					
ABC adherent	331 (5.9)	0.92 (0.78-1.09)	0.346	0.98 (0.83-1.17)	0.852
ABC non-adherent	250 (6.2)	Ref.		Ref.	
**TIA**					
ABC adherent	71 (1.3)	1.30 (0.88-1.93)	0.192	1.28 (0.85-1.92)	0.237
ABC non-adherent	38 (0.9)	Ref.		Ref.	
**HS**					
ABC adherent	83 (1.5)	0.37 (0.28-0.48)	<0.001	0.50 (0.38-0.66)	<0.001
ABC non-adherent	157 (3.9)	Ref.		Ref.	
**MI**					
ABC adherent	70 (1.2)	0.87 (0.61-1.23)	0.430	1.15 (0.79-1.66)	0.469
ABC non-adherent	56 (1.4)	Ref.		Ref.	
**HF**					
ABC adherent	124 (2.2)	0.52 (0.41-0.65)	<0.001	0.76 (0.60-0.97)	0.029
ABC non-adherent	165 (4.1)	Ref.		Ref.	
**Cardiovascular death**					
ABC adherent	60 (1.1)	0.43 (0.31-0.60)	<0.001	0.64 (0.46-0.90)	0.010
ABC non-adherent	97 (2.4)	Ref.		Ref.	
**All-cause mortality**					
ABC adherent	280 (5.0)	0.43 (0.37-0.50)	<0.001	0.72 (0.62-0.85)	<0.001
ABC non-adherent	455 (11.2)	Ref.		Ref.	

IS = ischaemic stroke; TIA = transient ischaemic attack; HS = haemorrhagic stroke; MI = myocardial infarction; HF = heart failure; HR = hazard ratio; CI = confidence interval.

Table S5. Characteristics of the inverse probability of treatment weighting cohort

	**ABC adherent (n=9,718)**	**ABC non-adherent (n=9,651)**	**SMD before IPTW**	**SMD after IPTW**
Age (years)	69.3±14.5	69.4±12.7	0.31	0.01
Male	5562.8 (57.2)	5522.1 (57.2)	0.09	<0.01
Smoking	3269.6 (33.6)	3262.2 (33.8)	0.11	<0.01
Alcohol	1912.0 (19.7)	1903.3 (19.7)	0.08	<0.01
NIHSS	6.4±4.8	6.2±4.9	0.34	0.02
Baseline comorbidities				
Atrial fibrillation	225.5 (2.3)	230.7 (2.4)	0.12	<0.01
Hypertension	826.9 (8.5)	810.3 (8.4)	0.05	<0.01
Ischaemic heart disease	157.8 (1.6)	158.3 (1.6)	0.06	<0.01
Diabetes mellitus	495.6 (5.1)	489.5 (5.1)	0.07	<0.01
Dyslipidaemia	266.1 (2.7)	252.3 (2.6)	0.03	<0.01
Chronic kidney disease	98.5 (1.0)	94.3 (1.0)	0.06	<0.01
Chronic liver disease	46.5 (0.5)	43.1 (0.4)	0.05	<0.01
Dementia	196.7 (2.0)	198.4 (2.1)	0.18	<0.01
Baseline medication use				
ACEi	1518.0 (15.6)	1516.3 (15.7)	0.04	<0.01
ARB	564.6 (5.8)	552.1 (5.7)	0.03	<0.01
Beta-blocker	2022.5 (20.8)	2029.9 (21.0)	0.13	<0.01
CCB	3013.7 (31.0)	3007.4 (31.2)	0.06	<0.01
Aspirin	1558.1 (16.0)	1559.6 (16.2)	0.16	<0.01
P2Y12 inhibitor	94.1 (1.0)	94.2 (1.0)	0.04	<0.01
Warfarin	111.9 (1.2)	114.7 (1.2)	0.11	<0.01
NOAC	73.0 (0.8)	81.0 (0.8)	0.08	0.01
Insulin	308.4 (3.2)	314.3 (3.3)	0.01	<0.01
Metformin	1340.0 (13.8)	1358.4 (14.1)	0.05	<0.01
Statin	1903.8 (19.6)	1876.8 (19.4)	0.14	<0.01

Values are shown as mean ± standard deviation or n (%).
NIHSS = National Institutes of Health Stroke Scale; ACEi = angiotensin-converting enzyme inhibitor; ARB = angiotensin receptor blocker; CCB = calcium channel blocker; NOAC = non-vitamin K antagonist oral anticoagulants; SMD = standardised mean difference; IPTW = inverse probability of treatment weighting.

Table S6. Sensitivity analysis for the effect of ABC_{stroke} criteria adherence on the risk for the composite outcome after inverse probability of treatment weighting

	**Unadjusted HR (95% CI)**	***P* value**	**Adjusted HR (95% CI)**	***P* value**
**Composite outcome**				
ABC adherent	0.72 (0.65-0.80)	<0.01	0.73 (0.66-0.80)	<0.01
ABC non-adherent	Ref.		Ref.	

HR = hazard ratio; CI = confidence interval.

Table S7. Baseline characteristics of patients with and without information of mRS or NIHSS recorded

	**Patients without information of mRS or NIHSS recorded (n=120,108)**	**Patients with information of mRS or NIHSS recorded (n=9,669)**	***P* value**
Age (years)	71.30 ±13.37	69.60 ±13.40	<0.001
Male	64,768 (53.9)	5,560 (57.5)	<0.001
Smoking	4,292 (3.6)	3,276 (33.9)	<0.001
Alcohol	2,186 (1.8)	1,908 (19.7)	<0.001
Baseline comorbidities			
Atrial fibrillation	3,074 (2.6)	222 (2.3)	0.121
Hypertension	12,714 (10.6)	812 (8.4)	<0.001
Ischaemic heart disease	2,464 (2.1)	154 (1.6)	0.002
Diabetes mellitus	7,497 (6.2)	498 (5.2)	<0.001
Dyslipidaemia	3,670 (3.1)	249 (2.6)	0.009
Chronic kidney disease	1,640 (1.4)	98 (1.0)	0.004
Chronic liver disease	799 (0.7)	49 (0.5)	0.073
Dementia	5,341 (4.4)	196 (2.0)	<0.001
Baseline medication use			
ACEi	21,650 (18.0)	1,543 (16.0)	<0.001
ARB	7,442 (6.2)	536 (5.5)	0.011
Beta-blocker	27,730 (23.1)	2,018 (20.9)	<0.001
CCB	43,616 (36.3)	3,016 (31.2)	<0.001
Aspirin	29,572 (24.6)	1,567 (16.2)	<0.001
P2Y12 inhibitor	1,778 (1.5)	90 (0.9)	<0.001
Warfarin	1,695 (1.4)	108 (1.1)	0.02
NOAC	983 (0.8)	70 (0.7)	0.349
Insulin	4,789 (4.0)	325 (3.4)	0.003
Metformin	19,235 (16.0)	1,370 (14.2)	<0.001
Statin	29,644 (24.7)	1,849 (19.1)	<0.001

Values are shown as mean ± standard deviation or n (%).

mRS = modified Rankin scale; NIHSS = National Institutes of Health Stroke Scale; ACEi = angiotensin-converting enzyme inhibitor; ARB = angiotensin receptor blocker; CCB = calcium channel blocker; NOAC = non-vitamin K antagonist oral anticoagulants.

Table S8. STROBE Statement—checklist of items that should be included in reports of observational studies

	Item No	Recommendation	Page No
**Title and abstract**	1	(*a*) Indicate the study's design with a commonly used term in the title or the abstract	Page 3 (Abstract)
		(*b*) Provide in the abstract an informative and balanced summary of what was done and what was found	Page 3 (Abstract)
**Introduction**			
Background/rationale	2	Explain the scientific background and rationale for the investigation being reported	Page 4 (Introduction)
Objectives	3	State specific objectives, including any prespecified hypotheses	Page 4 (End of Introduction)
**Methods**			
Study design	4	Present key elements of study design early in the paper	Page 5-9 (Methods)
Setting	5	Describe the setting, locations, and relevant dates, including periods of recruitment, exposure, follow-up, and data collection	Page 5-9 (Methods)
Participants	6	(*a*) *Cohort study*—Give the eligibility criteria, and the sources and methods of selection of participants. Describe methods of follow-up<br>*Case-control study*—Give the eligibility criteria, and the sources and methods of case ascertainment and control selection. Give the rationale for the choice of cases and controls<br>*Cross-sectional study*—Give the eligibility criteria, and the sources and methods of selection of participants	Page 5-9 (Methods) + Figure 1
		(*b*) *Cohort study*—For matched studies, give matching criteria and number of exposed and unexposed<br>*Case-control study*—For matched studies, give matching criteria and the number of controls per case	Page 5-9 (Methods) + Table S5
Variables	7	Clearly define all outcomes, exposures, predictors, potential confounders, and effect modifiers. Give diagnostic criteria, if applicable	Page 5-9 (Methods) + Table S1
Data sources/ measurement	8*	For each variable of interest, give sources of data and details of methods of assessment (measurement). Describe comparability of assessment methods if there is more than one group	Page 5-9 (Methods) + Table S1
Bias	9	Describe any efforts to address potential sources of bias	Page 5-9 (Methods) + Table S4-7
Study size	10	Explain how the study size was arrived at	Page 5-9 (Methods) + Figure 1

Quantitative variables	11	Explain how quantitative variables were handled in the analyses. If applicable, describe which groupings were chosen and why	Page 5-9 (Methods)
Statistical methods	12	(*a*) Describe all statistical methods, including those used to control for confounding	Page 5-9 (Methods)
		(*b*) Describe any methods used to examine subgroups and interactions	Page 8-9 (Methods)
		(*c*) Explain how missing data were addressed	N/A
		(*d*) *Cohort study*—If applicable, explain how loss to follow-up was addressed<br>*Case-control study*—If applicable, explain how matching of cases and controls was addressed<br>*Cross-sectional study*—If applicable, describe analytical methods taking account of sampling strategy	N/A
		(*e*) Describe any sensitivity analyses	Page 8-9 (Methods)

Continued on next page

**Results**			
Participants	13*	(a) Report numbers of individuals at each stage of study—eg numbers potentially eligible, examined for eligibility, confirmed eligible, included in the study, completing follow-up, and analysed	Figure 1
		(b) Give reasons for non-participation at each stage	Figure 1
		(c) Consider use of a flow diagram	Figure 1
Descriptive data	14*	(a) Give characteristics of study participants (eg demographic, clinical, social) and information on exposures and potential confounders	Page 9 (Results) + Table 1 + Table S2
		(b) Indicate number of participants with missing data for each variable of interest	N/A
		(c) *Cohort study*—Summarise follow-up time (eg, average and total amount)	Page 10 (Results)
Outcome data	15*	*Cohort study*—Report numbers of outcome events or summary measures over time	Page 9-12 (Results) + Figure 2-3 + Table 2-3
		Case-control study—Report numbers in each exposure category, or summary measures of exposure	N/A
		Cross-sectional study—Report numbers of outcome events or summary measures	N/A
Main results	16	(*a*) Give unadjusted estimates and, if applicable, confounder-adjusted estimates and their precision (eg, 95% confidence interval). Make clear which confounders were adjusted for and why they were included	Page 9-12 (Results) + Table 2-3 + Table S3,S4,S6
		(*b*) Report category boundaries when continuous variables were categorized	N/A
		(*c*) If relevant, consider translating estimates of relative risk into absolute risk for a meaningful time period	N/A
Other analyses	17	Report other analyses done—eg analyses of subgroups and interactions, and sensitivity analyses	Page 11-12 (Results) + Figure 4 + Table S4-S6
**Discussion**			
Key results	18	Summarise key results with reference to study objectives	Page 12 (Discussion)
Limitations	19	Discuss limitations of the study, taking into account sources of potential bias or imprecision. Discuss both direction and magnitude of any potential bias	Page 15-16 (Limitations)
Interpretation	20	Give a cautious overall interpretation of results considering objectives, limitations, multiplicity of analyses, results from similar studies, and other relevant evidence	Page 12-16 (Discussion)
Generalisability	21	Discuss the generalisability (external validity) of the study results	Page 12-16 (Discussion)
**Other information**			
Funding	22	Give the source of funding and the role of the funders for the present study and, if applicable, for the original study on which the present article is based	Page 17

*Give information separately for cases and controls in case-control studies and, if applicable, for exposed and unexposed groups in cohort and cross-sectional studies.

Note: An Explanation and Elaboration article discusses each checklist item and gives methodological background and published examples of transparent reporting. The STROBE checklist is best used in conjunction with this article (freely available on the Web sites of PLoS Medicine at http://www.plosmedicine.org/, Annals of Internal Medicine at http://www.annals.org/, and Epidemiology at http://www.epidem.com/). Information on the STROBE Initiative is available at www.strobe-statement.org.
